# Baseline characteristics and 2-year functional outcome data of patients undergoing an arthroscopic rotator cuff repair in Switzerland, results of the ARCR_Pred study

**DOI:** 10.1371/journal.pone.0316712

**Published:** 2025-01-10

**Authors:** Thomas Stojanov, Laurent Audigé, Soheila Aghlmandi, Claudio Rosso, Philipp Moroder, Thomas Suter, Mai Lan Dao Trong, Emanuel Benninger, Beat Moor, Christophe Spormann, Holger Durchholz, Gregory Cunningham, Alexandre Lädermann, Michael Schär, Matthias Flury, Karim Eid, Markus Scheibel, Christian Candrian, Bernhard Jost, Matthias A. Zumstein, Karl Wieser, David Schwappach, Sabina Hunziker, ARCR_Pred Study Group, Andreas M. Müller

**Affiliations:** 1 Orthopaedic Surgery and Traumatology, University Hospital Basel, Basel, Switzerland; 2 Surgical Outcome Research Center, University Hospital of Basel, Basel, Switzerland; 3 Division of Clinical Epidemiology, Department of Clinical Research, University Hospital Basel and University of Basel, Basel, Switzerland; 4 Research and Development, Schulthess Klinik, Zürich, Switzerland; 5 ARTHRO Medics Ltd, Shoulder and Elbow Center, Basel, Switzerland; 6 Department of Shoulder and Elbow Surgery, Center for Musculoskeletal Surgery, Charité Medicine University, Berlin, Germany; 7 Shoulder and Elbow Surgery, Schulthess Klinik, Zürich, Switzerland; 8 Orthopaedic Shoulder and Elbow, Cantonal Hospital Baselland, Bruderholz, Switzerland; 9 Orthopaedic Surgery and Traumatology, Public Hospital Solothurn, Solothurn, Switzerland; 10 Orthopaedic Surgery and Traumatology, Winterthur Cantonal Hospital, Winterthur, Switzerland; 11 Service for Orthopaedics and Traumatology of the Musculoskeletal System, Valais Hospital Center, Martigny, Switzerland; 12 Center for Endoprosthetics and Joint Surgery, Endoclinic, Zürich, Switzerland; 13 Klinik Gut Sankt Moritz, Saint Moritz, Switzerland; 14 Shoulder Center, Hirslanden Clinique La Colline, Geneva, Switzerland; 15 Division of Orthopaedics and Trauma Surgery, Department of Surgery, Geneva University Hospitals, Geneva, Switzerland; 16 Division of Orthopaedics and Trauma Surgery, La Tour Hospital, Meyrin, Switzerland; 17 FORE Foundation for Research and Teaching in Orthopedics, Sports Medicine, Trauma, and Imaging in the Musculoskeletal System, Meyrin, Switzerland; 18 Department of Orthopaedic Surgery and Traumatology, Inselspital, Bern University Hospital, University of Bern, Bern, Switzerland; 19 Center for Orthopaedics and Neurosurgery, In-Motion, Wallisellen, Switzerland; 20 Clinic for Orthopaedics and Traumatology, Baden Cantonal Hospital, Baden, Switzerland; 21 Trauma and Ortho Unit, Lugano Regional Hospital, Lugano, Switzerland; 22 Clinic for Orthopaedic Surgery and Traumatology of the Musculoskeletal System, Cantonal Hospital of St.Gallen, St Gallen, Switzerland; 23 Shoulder, Elbow and Orthopaedic Sports Medicine, Orthopaedics Sonnenhof, Bern, Switzerland; 24 Stiftung Lindenhof, Campus SLB, Swiss Institute for Translational and Entrepreneurial Medicine, Bern, Switzerland; 25 Department of Orthopaedics, Balgrist University Hospital, University of Zurich, Zürich, Switzerland; 26 Institute of Social and Preventive Medicine, University of Bern, Bern, Switzerland; 27 Medical Communication/Psychosomatic Medicine, University Hospital Basel, Basel, Switzerland; Carol Davila University of Medicine and Pharmacy: Universitatea de Medicina si Farmacie Carol Davila din Bucuresti, ROMANIA

## Abstract

The ARCR_Pred study was initiated to document and predict the safety and effectiveness of arthroscopic rotator cuff repair (ARCR) in a representative Swiss patient cohort. In the present manuscript, we aimed to describe the overall and baseline characteristics of the study, report on functional outcome data and explore case-mix adjustment and differences between public and private hospitals. Between June 2020 and November 2021, primary ARCR patients were prospectively enrolled in a multicenter cohort across 18 Swiss and one German orthopedic center. Baseline characteristics, including sociodemographic and diagnostic variables, were reported. Clinical scores and patient-reported outcome measures were assessed up to 24-month follow-up. After screening 2350 individuals, 973 patients with ARCR were included. Follow-up rates reached 99%, 95%, 89% and 88% at 6 weeks, 6, 12, and 24 months, respectively. While the proportion of massive tears was higher in the study population (44% vs. 20%, Std. Diff. = 0.56), there were no other major differences in key characteristics between enrolled and non-enrolled patients or in patients lost to follow-up. Functional scores improved over time, with positive changes rates ranging from 83% to 92% at 6-month, reaching 91% to 97% at 12- and 24-month follow-up. In linear mixed models, used to estimate the associations between baseline factors, hospital type and standardized 0–100 scores, marginal effects for time ranged from 20 to 30, 28 to 39 and 34 to 41 points at the 6-, 12- and 24-month follow-up, respectively. Except at the 12-month follow-up, where marginal effects for the interaction terms ranged from -5 to -4 points in the standardized scores, there were no consistent outcome differences between public and private hospitals. Increasing number of years of education was consistently associated with better scores, greater feelings of depression and anxiety, smoking and ASA group III-IV were consistently associated with worse scores. Tear severity showed a consistent negative association solely for the Constant-Score. The ARCR_Pred study shows high potential for generalizability to the population of patients undergoing an ARCR in Switzerland. Further analyses are needed to establish relevant clinimetrics for the Swiss population and to compare outcomes for surgical techniques, surgeon experiences profiles and post-operative management.

## Introduction

Affecting more than one in five persons in the general population, rotator cuff disease is one of the most common musculoskeletal disorders [1–3]. When conservative treatment fails, arthroscopic rotator cuff repair (ARCR) is considered to reduce pain and restore function [4]. With the recent increasing number of orthopedic surgeries, coupled with their associated costs, ARCR constitute a significant burden for healthcare systems [5]. Furthermore, the variability in reported adverse events rates suggests inconsistent patient benefits from ARCR procedures [6, 7]. Identification of patients likely to benefit from ARCR in functional or safety outcomes could support decision-making for patients and surgeons [8]. Prediction models, relying on sound development and high-quality prospective studies, offer valuable support by combining predictor values for risk communication and treatment decisions [9]. In the orthopedic field, especially for ARCR, such high-quality studies are lacking [10–13].

Complication rates and patient profiles were also shown to differ between public and private healthcare providers in other settings [14]. In Switzerland, patient hospital (or specialist) choice is usually influenced by a combination of factors [15], including the recommendations of general practitioners and social networks [16] and the subscription to additional health insurance [17]. Indeed, Switzerland has compulsory basic health insurance coverage, with voluntary (semi-) private insurance plans covering additional services [18], typically used by higher socioeconomic groups [17, 19]. In the field of ARCR, Australian researchers reported that care setting type (public *vs*. private) might be a potential prognostic factor for repair integrity [20]. Data describing the distribution of baseline case-mix variables across hospital type and their association with ARCR outcomes is lacking in Switzerland.

In this context, we initiated the ARCR_Pred study in June 2020 in 19 public and private hospitals [21]. Focused on a Swiss multicenter prospective ARCR cohort, the study documented patient-, diagnostic-, operation- and post-operative management-related factors. Outcome parameters encompass various clinical scores, patient-reported outcomes measures (PROMs), adverse events, activities of daily living, and quality of life scores up to two years after the initial ARCR.

The aim of this paper was to present the overall baseline key characteristics of the ARCR_Pred study, report overall two-year patient functional outcome data, explore differences in key characteristics distributions and outcomes between public and private hospitals.

## Methods

Items included in the present manuscript are reported according to the STROBE guidelines for cohort studies [22]. Ethical approval was obtained on April 1^st^, 2020, from the lead ethics committee (EKNZ, Basel Switzerland; ID: 2019–02076, trial registration number NCT04321005). All participants provided informed written consent before enrollment in the study [21].

### Study design and setting

**Fig 1:**
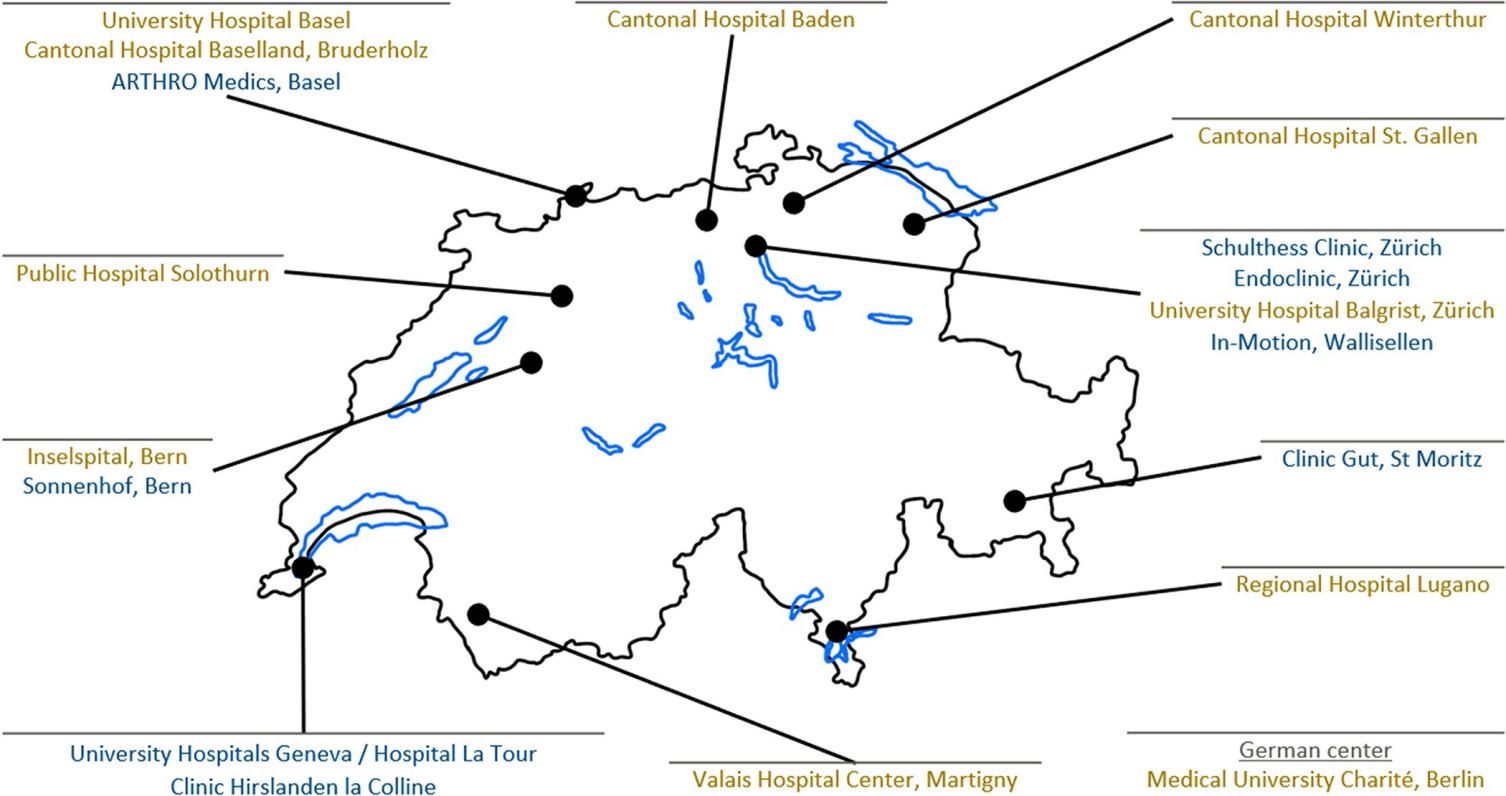
Geographical distribution of ARCR_Pred cohort study centers. **Note**: Yellow and blue text indicate public and private hospital study centers, respectively. Study centers located in the same city are listed alphabetically.

Patients were recruited from June 2020 to November 2021 and followed up two years after the ARCR across 18 Swiss centers and one in Germany (Fig 1), eight (44%) of which were private centers. A sample size of 970 patients was predetermined to meet methodological requirements for prediction model development for the study primary outcomes of occurrence of shoulder stiffness and achievement of shoulder function using the Oxford Shoulder Score (OSS). Additional details are available in the study protocol [21].

### Enrollment of study participants

#### Inclusion criteria

All adult patients were considered for inclusion if they were diagnosed with a partial or full-thickness rotator cuff tear by magnetic resonance imaging (MRI), planned for a primary arthroscopic surgical repair, and gave their written informed consent to participate in the cohort study.

#### Exclusion criteria

Patients undergoing a specific surgical procedure for irreparable tears (i.e., tendon transfer, subacromial spacer or superior capsular reconstruction), revision operations, and open or mini-open reconstructions were excluded. Patients not fluent in German, French, Italian or English, and pregnant women were excluded. Patients undergoing bilateral ARCR were included only for their first intervention.

#### Screening log

Patients who were eligible for the study over the enrollment period of each center—defined as the time between the first signed informed consent and the last surgery—were documented in a screening log including age at surgery, sex, and tear severity (partial tear, single full-thickness tear, two or three tendons tears with only one full-thickness tear or massive tear with two or more full-thickness tears, as defined by Gerber et al. [23]).

### Follow-up data

Patients were asked to undergo a baseline evaluation no more than 2 months before the date of surgery, followed by clinical follow-ups at 6 weeks (±1 week), 6 (±1 month) and 12 (±1 month) postoperatively. Patient questionnaires were administered at baseline, and at 6 (±1 month), 12 (±1 month), and 24 (±2 months) months post-surgery.

### Study variables and data management

#### Data capture and management

An electronic data capture system database was designed with REDCap [24], which allowed on-site data entry and remote central data monitoring. Baseline MRI and radiographic images were coded and centralized. Patients were invited to provide their e-mail address upon patient informed consent for the online completion of all study-related questionnaires and to receive informative newsletters.

### Study variables

The following patient-level variables were selected for key characteristics description: age at surgery, sex, body mass index (BMI), smoking status, alcohol use, presence of comorbidities including diabetes, American Society of Anesthesiologists (ASA) classification [25], duration of patient-reported symptoms, level of depression and anxiety using one question from the EQ-5D-5L [26] quality of life instrument, number of years of education, baseline level of sports activities, tear onset type (traumatic *vs*. degenerative) and pre-operative treatment (steroid infiltrations, medication, and physiotherapy). The highest degree of fatty infiltration of repaired tendons based on the Goutallier classification was reported relying on baseline MRI [27]. Diagnostic-related variables such as rotator cuff tear severity and individual rotator cuff tendons integrity (intact, partial or complete tear for the supraspinatus (SSP), infraspinatus (ISP), subscapularis (SSC)) were based on intra-operative findings.

#### Clinical score and PROMs

We reported and analyzed four functional outcomes: (1) the Constant score, ranging from 0 (worse function) to 100 (best function) points [28] (a clinical outcome), (2) the OSS, ranging from 0 (worse function) to 48 (best function) points (a patient-reported outcome (PRO), the main ARCR_Pred study endpoint [29]), (3) the Subjective Shoulder Value (SSV) (a PRO), ranging from 0% (worse function) to 100% (best function) [30]; and the level of pain using a numeric rating scale (NRS) ranging from 0 (no pain) to 10 (intolerable pain) points. Positive changes in scores were defined as the difference between the score value at a given time point and the baseline score value.

### Statistical methods

#### Descriptive statistics

Categorical and numeric variables were described as numbers (N) with percentages (%) and mean values with SD (Min—Max), respectively. The standardized difference (Std. Diff.) was used to compare the imbalance between enrolled and non-enrolled patients; and between patients with follow-up and those lost-to-follow-up [31, 32]. For the comparison between patients with follow-up and those lost-to-follow-up, we compared the last recorded OSS value (e.g. for patients lost-to-follow-up at 12 and 24 months, we compared the 6- and 12-month OSS between lost and documented patient groups, respectively). To compare outcome data between public and private hospitals, we reported mean differences.

#### Modeling 2-year outcome data

Considering the hierarchical and longitudinal structure of the ARCR_Pred data, we used linear mixed models to model outcomes data. A linear mixed model was constructed for the four outcome variables (note that Constant-Score was collected solely at baseline, 6- and 12-month follow-up) using complete-case data. Data were clustered at two levels (center-level and patient-level with repeated outcome measures (baseline, 6-, 12- and 24-month)). Random intercepts were then estimated at both the clinic- and the patient-level. For interpretability purposes, the pain score was reversed, so that a higher pain score indicated a better outcome. All outcome values were then rescaled onto a 0–100 scale. Model coefficients could then be interpreted equally across outcomes.

In the context of the present analysis, solely baseline variables were included in the final model to adjust for confounding. Three study authors chose the variables to adjust for (TS, LA, AMM), representing the case-mix profile most likely to influence outcomes. Differences in variables distributions across hospital type and the results of a recent systematic review summarizing the evidence related to prognostic factors associated with post-operative functional outcomes guided their choice [13]. In each model, we added an interaction term between time and the hospital type. This way, we could assess whether the association between hospital type and the outcomes depended on a specific timepoint.

Overall apparent model performances were then assessed and reported using conditional and marginal R2, which represent the percentage of variation explained by both the random and fixed effects or by fixed effects only, respectively.

## Results

### Patient selection and follow-up

#### Screening data

**Fig 2:**
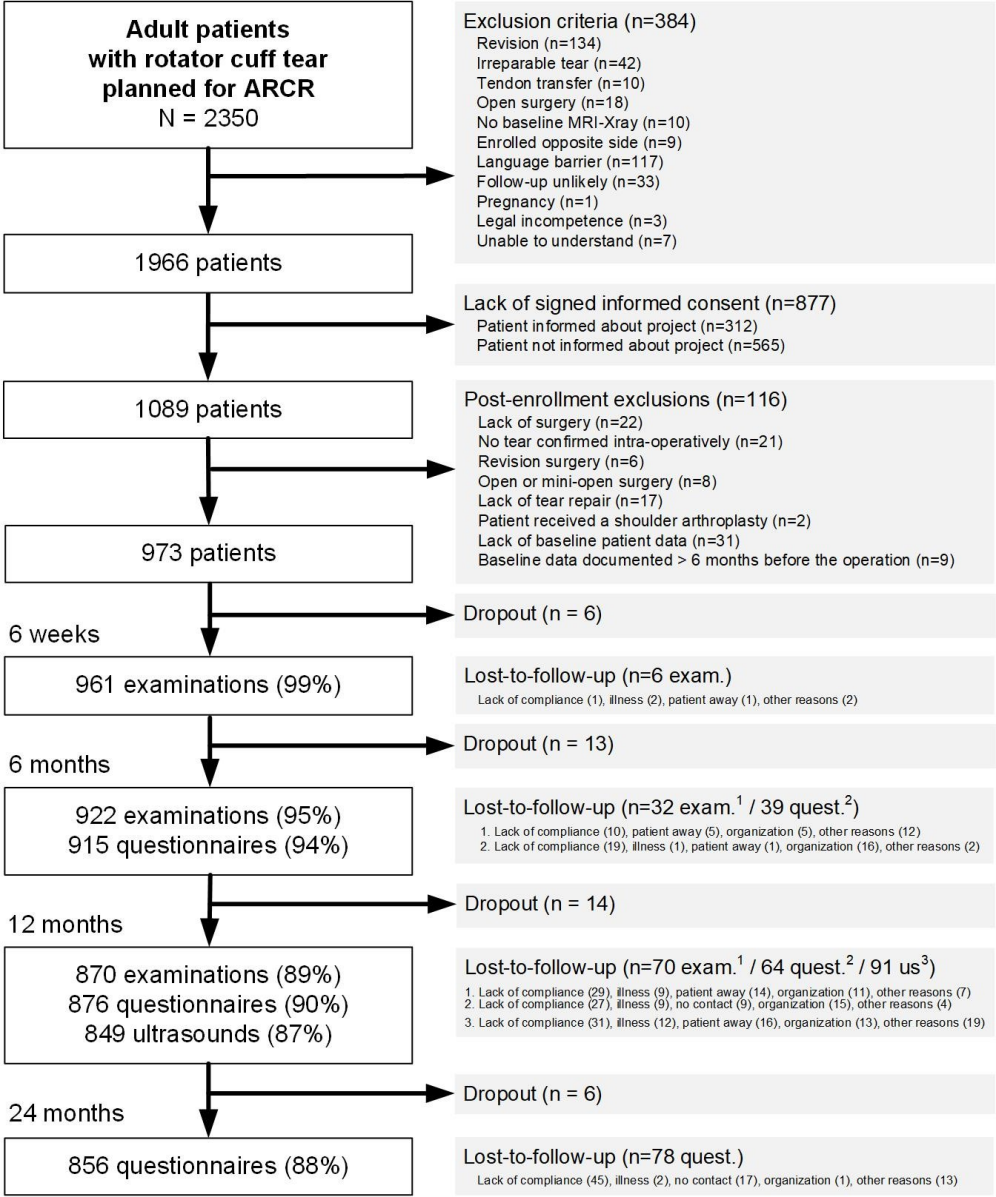
Patient selection flowchart and follow-up. This figure presents the ARCR_Pred patient selection and follow-up flow. Abbreviations: ARCR: Arthroscopic Rotator Cuff Repair; MRI: Magnetic Resonance Imaging.

Out of 2,350 screened patients, 1,089 provided consent. Post-enrollment eligibility criteria exclusions left 973 patients (51.5% of 1,890 eligible) in the study (Fig 2). While tear severity varied, with fewer partial (15% *vs*. 25%) and two or three tendons (15% *vs*. 26%) but more massive tears (44% *vs*. 20%) in the population enrolled in study (Std. Diff. = 0.56), there were no major differences in age and sex distribution. S1 Table provides additional information on the distribution comparison of key characteristics between enrolled and non-enrolled patients.

#### Follow-up data

Clinical examinations achieved follow-up rates of 99%, 95%, and 89% at 6 weeks, 6 months, and 12 months respectively (Fig 2). Regarding questionnaires, follow-up rates were 94%, 90%, and 88% at 6-, 12- and 24-month. S2 Table presents additional information comparing key characteristics distribution between patients lost to follow-up and those retained in the study. Lost-to-follow-up patients had on average 2 points less in the last known OSS value (Std. Diff. ranged between 0.25 and 0.36). No other differences could be identified. Fig 2 provides reasons for lost-to-follow-up.

### Baseline characteristics

**Fig 3:**
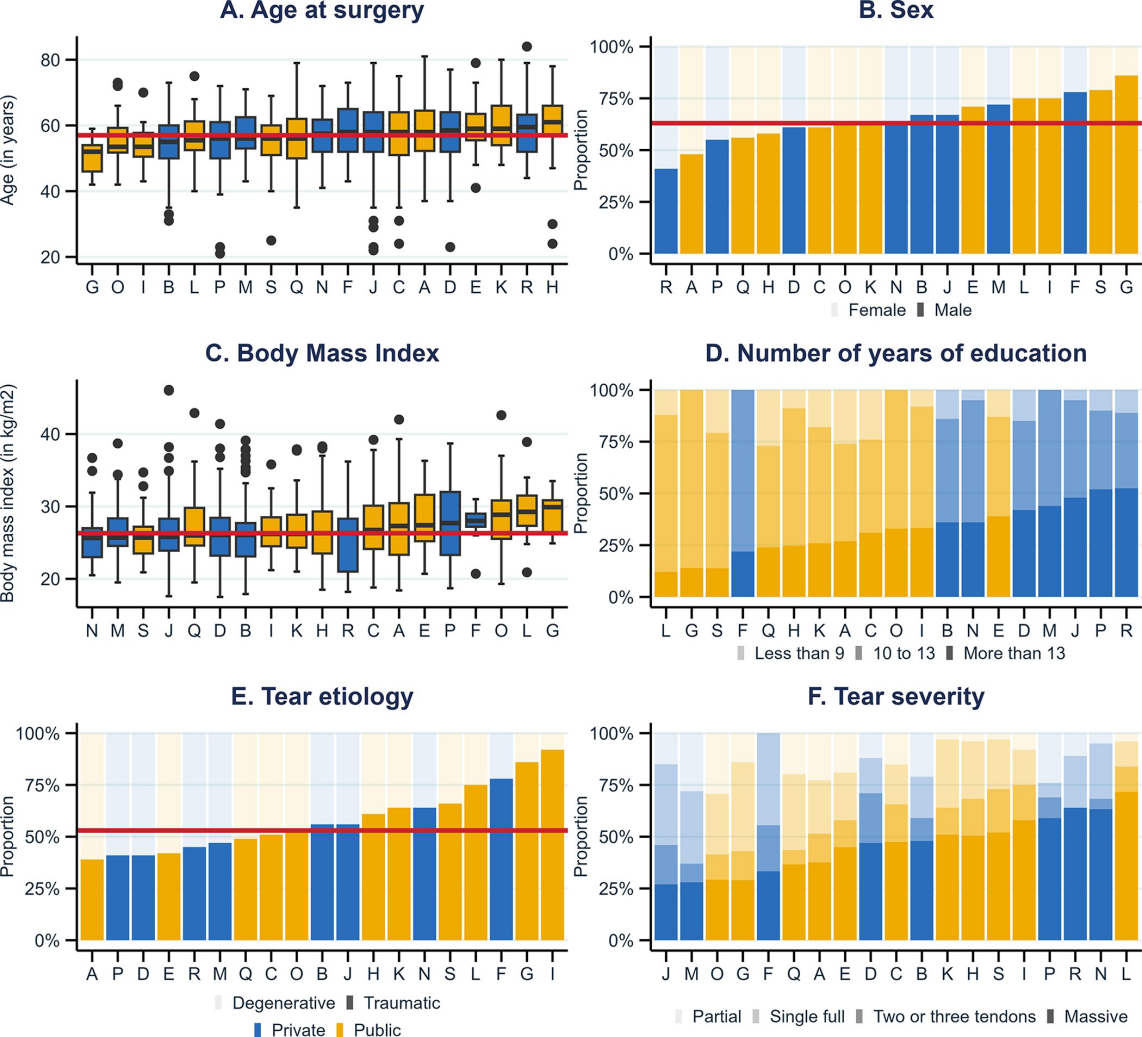
Distribution of key baseline characteristics across study centers. This figure shows the distribution of six baseline characteristics across the 19 study centers. Red lines correspond to: (A) overall median age (57 years); (B) overall proportion of males (63%); (C) overall median BMI (26.4 kg/m^2^) and (E) overall proportion of traumatic tears (53%).

Enrolled patients averaged 57 years (SD = 9), predominantly male (63%), with an average BMI of 26.8 kg/m2 (Table 1). The rate of current smokers was slightly more common in public hospitals (Std. Diff. = 0.23). While most were either ASA I (44%) or ASA II (49%), public hospitals had fewer ASA I (36% *vs*. 51%) and more ASA III-IV patients (11% *vs*. 4.7%) (Std. Diff. = 0.34). The proportion of patients with more than 13 years of education was higher in private hospitals (44% *vs*. 27%, Std. Diff. = 0.39). The proportion of full-thickness ISP tears was higher in private hospitals (22% *vs*. 11%, Std. Diff. = 0.31). According to study center, median age and BMI ranged from 52 to 61 years old and 17.5 to 46.1 kg/m^2^, respectively (Fig 3).

**Table 1:** Baseline characteristics.

		Hospital type		
Characteristic	Overall, N = 973	Private, N = 530	Public, N = 443	Difference^1^
**Age at surgery**	57 (9; 21–84)	57 (10; 21–84)	58 (9; 24–81)	-0.09
**Male sex**	611 (63%)	337 (64%)	274 (62%)	0.04
**BMI (in kg/m** ^**2**^ **)**	26.8 (4.4; 17.5–46.1)	26.3 (4.4; 17.5–46.1)	27.4 (4.4; 18.4–42.9)	-0.25
**Current smoker**	204 (21%)	89 (17%)	115 (26%)	0.23
**Alcohol use**				0.08
*No*	232 (24%)	125 (24%)	107 (24%)	
*Occasionally*	645 (66%)	358 (68%)	287 (65%)	
*At least daily*	96 (9.9%)	47 (8.9%)	49 (11%)	
**One or more comorbidities**	511 (53%)	275 (52%)	236 (53%)	0.03
**Diabetes**	43 (4.4%)	20 (3.8%)	23 (5.2%)	0.07
**ASA Classification**				0.34
*I*	429 (44%)	269 (51%)	160 (36%)	
*II*	472 (49%)	236 (45%)	236 (53%)	
*III-IV*	72 (7.4%)	25 (4.7%)	47 (11%)	
**Patient-reported symptom duration**				0.24
*1 week or less*	19 (2.0%)	13 (2.5%)	6 (1.4%)	
*>1 week to 1 month*	110 (11%)	71 (13%)	39 (8.8%)	
*>1 to 3 months*	203 (21%)	115 (22%)	88 (20%)	
*>3 to 6 months*	166 (17%)	89 (17%)	77 (17%)	
*>6 months to 1 year*	173 (18%)	76 (14%)	97 (22%)	
*>1 year*	302 (31%)	166 (31%)	136 (31%)	
**Level of anxiety and depression (EQ-5D-5L)**				0.08
*Not anxious or depressed*	607 (62%)	339 (64%)	268 (60%)	
*Slightly anxious or depressed*	248 (25%)	132 (25%)	116 (26%)	
*At least moderately anxious or depressed*	118 (12%)	59 (11%)	59 (13%)	
**Baseline level of sports activity**				0.18
*Never*	221 (23%)	111 (21%)	110 (25%)	
*Less than once a week*	95 (9.8%)	51 (9.6%)	44 (9.9%)	
*Once a week*	173 (18%)	84 (16%)	89 (20%)	
*Twice a week or more*	484 (50%)	284 (54%)	200 (45%)	
**Traumatic tear**	516 (53%)	272 (51%)	244 (55%)	0.08
**Number of years of education**				0.39
*Up to 9 years of education*	129 (13%)	49 (9.2%)	80 (18%)	
*10 to 13 years of education*	493 (51%)	250 (47%)	243 (55%)	
*13+ years of education*	351 (36%)	231 (44%)	120 (27%)	
**Pre-operative steroid infiltrations**	290 (30%)	144 (27%)	146 (33%)	0.13
**Pre-operative medication**	409 (42%)	242 (46%)	167 (38%)	0.16
**Pre-operative physiotherapy**	387 (40%)	190 (36%)	197 (44%)	0.18
**Highest degree of fatty infiltration for repaired tendons**				0.27
*Stage 0*	498 (51%)	300 (57%)	198 (45%)	
*Stage 1*	338 (35%)	172 (32%)	166 (37%)	
*Stage 2*	123 (13%)	54 (10%)	69 (16%)	
*Stage 3–4*	14 (1.4%)	4 (0.8%)	10 (2.3%)	
**Rotator cuff tear severity (intra-operative)**				0.1
*Partial tear*	147 (15%)	87 (16%)	60 (14%)	
*Single full tear*	255 (26%)	143 (27%)	112 (25%)	
*Two or three tendons (only one full)*	143 (15%)	77 (15%)	66 (15%)	
*Massive tear*	428 (44%)	223 (42%)	205 (46%)	
**Supraspinatus tendon integrity**				0.11
*Intact tendon*	72 (7.4%)	33 (6.2%)	39 (8.8%)	
*Partial tear*	177 (18%)	102 (19%)	75 (17%)	
*Complete tear*	724 (74%)	395 (75%)	329 (74%)	
**Infraspinatus tendon integrity**				0.1
*Intact tendon*	573 (59%)	302 (57%)	271 (61%)	
*Partial tear*	106 (11%)	57 (11%)	49 (11%)	
*Complete tear*	294 (30%)	171 (32%)	123 (28%)	
**Subscapularis tendon integrity**				0.27
*Intact tendon*	513 (53%)	305 (58%)	208 (47%)	
*Partial tear*	125 (13%)	73 (14%)	52 (12%)	
*Complete tear*	335 (34%)	152 (29%)	183 (41%)	

Mean (SD; Min—Max); n (%)

### Functional scores (Fig 4)

**Fig 4:**
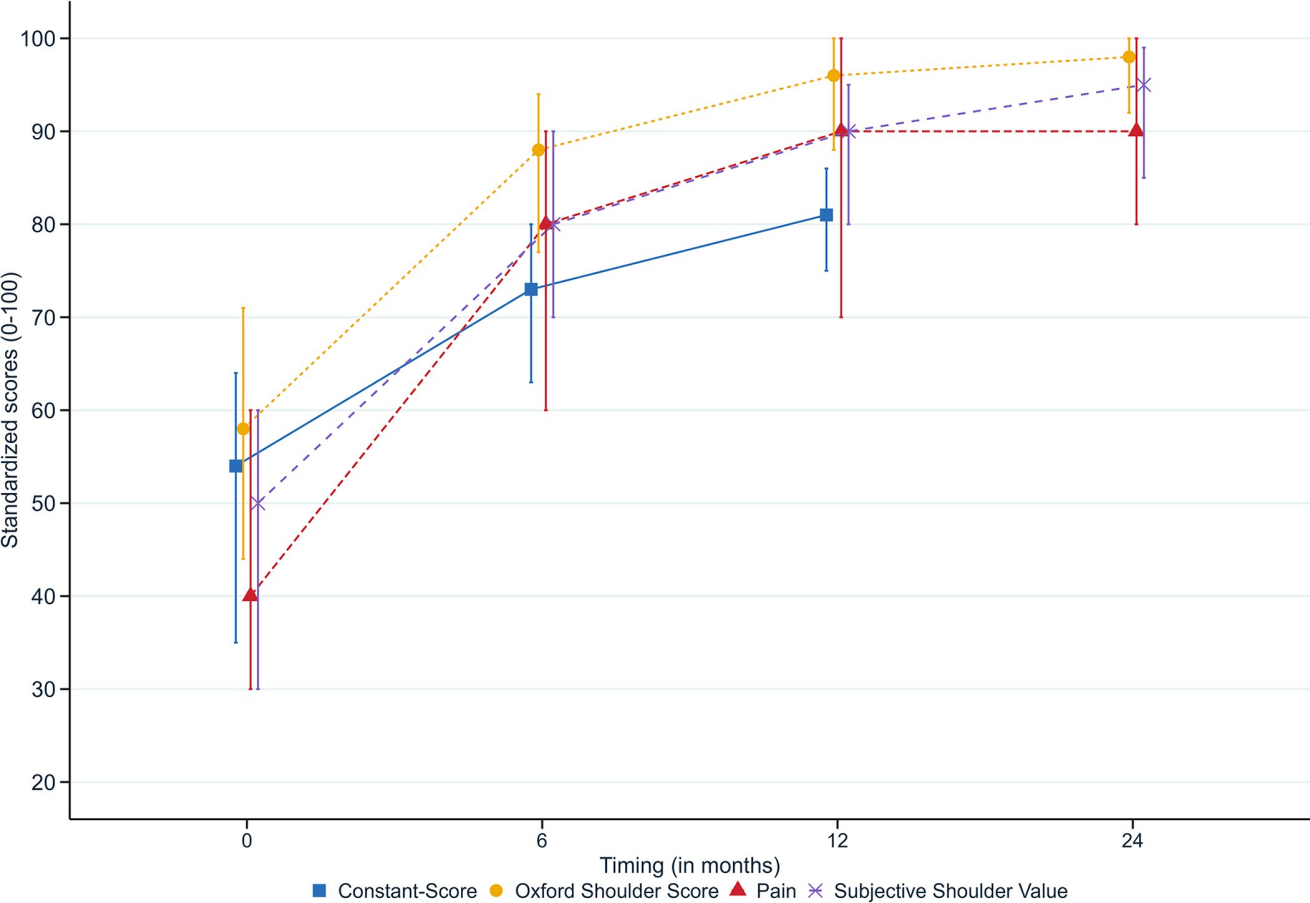
Patients functional scores. This figure shows median (dots), 25^th^ and 75^th^ quantile (error bars) of rescaled score values (on a 0–100 scale) over time.

Among patients with available follow-up data, the proportion of patients with positive change scores at 6-month was 83% (739/888), 92% (843/914), 84% (770/914), and 86% (786/913) for the CS, the OSS, Pain and the SSV, respectively. At 12-month follow-up, positive change scores rates were 94% (800/847), 97% (849/876), 91% (800/876), 96% (821/855). At 24-month follow-up, rates were 96% (820/854) for the OSS, 91% (782/856) for pain and 94% (808/855) for the SSV. S3 Table provides additional information on outcomes values and their standard deviation as well as on the comparison between baseline and post-operative score values. Private hospitals tended to have slightly better outcomes (differences ranged between 1 and 5 points, depending on the scale) while baseline scores were similar.

### Associations between baseline characteristics and functional scores

After transformation of scores on a 0–100 scale and adjustment for a large set of baseline variables (Table 2), marginal effects for time ranged from 20 to 30, 28 to 39 and 34 to 41 points at the 6-, 12- and 24-month follow-up, respectively. Except at the 12-month follow-up, where marginal effects for the interaction terms ranged from -5 to -4 points in the standardized scores, there were no consistent outcome differences between public and private hospital.

**Table 2:** Linear mixed effect modelling results.

Parameter	Constant-Score	Oxford Shoulder Score	Pain	Subjective Shoulder Value
**Fixed effects**				
**Time**				
*Baseline (intercept)*	61.2 (53.7 to 68.6)	54.1 (47.4 to 60.8)	27.9 (18.9 to 36.8)	40.9 (33.8 to 47.9)
*6-month*	20.3 (18.8 to 21.8)	25.2 (23.8 to 26.6)	29.5 (27.5 to 31.6)	29 (27.2 to 30.7)
*12-month*	28.5 (26.9 to 30)	32.5 (31.1 to 33.9)	39.1 (36.9 to 41.2)	38.8 (37.1 to 40.6)
*24-month*	NA	34 (32.6 to 35.4)	39.6 (37.5 to 41.7)	40.7 (38.9 to 42.4)
**Hospital type**				
*Private*	Ref.	Ref.	Ref.	Ref.
*Public*	-0.1 (-3.5 to 3.2)	1.2 (-1.5 to 3.9)	1 (-2 to 4)	0.3 (-2.4 to 2.9)
**Interaction term**				
*6-month x Public*	-5.4 (-7.7 to -3.2)	-2 (-4 to 0.1)	-0.6 (-3.7 to 2.5)	-2.9 (-5.5 to -0.4)
*12-month x Public*	-4.4 (-6.7 to -2.2)	-3.3 (-5.4 to -1.2)	-4.2 (-7.3 to -1)	-4.4 (-7 to -1.8)
*24-month x Public*	NA	-2.3 (-4.4 to -0.2)	-0.1 (-3.3 to 3)	-2.9 (-5.5 to -0.3)
**Age (year-unit)**	-0.1 (-0.2 to -0.1)	0.1 (0 to 0.2)	0.2 (0.1 to 0.3)	0.1 (0 to 0.2)
**Sex**				
*Female*	Ref.	Ref.	Ref.	Ref.
*Male*	3.2 (1.7 to 4.8)	2.1 (0.7 to 3.5)	3.1 (1.2 to 4.9)	-1.3 (-2.8 to 0.1)
**Body mass index (kg/m2-unit)**	-0.2 (-0.4 to -0.1)	-0.2 (-0.4 to -0.1)	-0.2 (-0.5 to 0)	-0.1 (-0.3 to 0.1)
**Smoking status**				
*Not current smoker*	Ref.	Ref.	Ref.	Ref.
*Current smoker*	-2.6 (-4.4 to -0.8)	-1.5 (-3.1 to 0.2)	-2.1 (-4.3 to 0.1)	-1.2 (-2.9 to 0.5)
**Alcohol consumption**				
*No*	Ref.	Ref.	Ref.	Ref.
*Occasionally*	2.5 (0.7 to 4.2)	1.3 (-0.4 to 2.9)	1.6 (-0.5 to 3.8)	0.9 (-0.8 to 2.5)
*At least daily*	0.1 (-2.7 to 2.9)	-0.5 (-3 to 2)	0 (-3.4 to 3.4)	-0.2 (-2.9 to 2.4)
**ASA Classification**				
*I*	Ref.	Ref.	Ref.	Ref.
*II*	0.3 (-1.3 to 2)	-0.7 (-2.2 to 0.7)	-1.3 (-3.2 to 0.7)	-0.2 (-1.8 to 1.3)
*III-IV*	-3 (-6.1 to 0.1)	-3 (-5.8 to -0.2)	-2.3 (-6.1 to 1.5)	-1.8 (-4.7 to 1.2)
**Anxiety and depression (EQ-5D-5L question)**				
*Not anxious or depressed*	Ref.	Ref.	Ref.	Ref.
*Slightly anxious or depressed*	-5.4 (-6.9 to -3.9)	-7.5 (-8.7 to -6.3)	-8.8 (-10.6 to -6.9)	-7.4 (-8.9 to -5.9)
*At least moderately anxious or depressed*	-11.3 (-13.5 to -9.2)	-17 (-18.8 to -15.2)	-16.3 (-18.9 to -13.6)	-14.8 (-17 to -12.7)
**Number of years of education**				
*Up to 9*	Ref.	Ref.	Ref.	Ref.
*10 to 13*	4.4 (2.2 to 6.6)	5.3 (3.3 to 7.3)	9.2 (6.4 to 11.9)	4.4 (2.2 to 6.5)
*13+*	7.2 (4.9 to 9.6)	8 (5.8 to 10.1)	12.2 (9.2 to 15.1)	7.4 (5.2 to 9.7)
**Tear severity**				
*Partial tear*	Ref.	Ref.	Ref.	Ref.
*Single full tear*	-0.9 (-3.2 to 1.4)	1.4 (-0.7 to 3.5)	4.4 (1.5 to 7.2)	1.8 (-0.4 to 4)
*Two or three tendons (only one full)*	-1.6 (-4.2 to 1.1)	0.2 (-2.2 to 2.6)	2.8 (-0.4 to 6.1)	0.9 (-1.7 to 3.4)
*Massive tear*	-2.3 (-4.5 to -0.1)	0.7 (-1.3 to 2.7)	4.8 (2.1 to 7.5)	2.1 (0 to 4.2)
**RANDOM INTERCEPTS**				
Patient-level standardized deviation	8.1	8.1	10.4	7.6
Clinic-level standardized deviation	2.8	2.1	1.5	1.6
Residual standardized deviation	12.2	11.2	16.9	14
**Apparent model performance**				
*R2 (conditional)*	0.63	0.72	0.62	0.66
*R2 (marginal)*	0.45	0.56	0.48	0.55

This table presents the fixed effects estimated from a multilevel regression analysis assessing the association between various factors on shoulder-related outcomes over time. The parameters include the Constant-Score, Oxford Shoulder Score, Pain, and Subjective Shoulder Value. Interaction terms between time and hospital type are included to evaluate differences between private and public hospital settings at specific timepoints. Note that Constant-Score was not collected at 24-month. Additionally, patient characteristics such as age, sex, body mass index (BMI), smoking status, alcohol consumption, American Society of Anesthesiologists (ASA) classification, anxiety and depression levels, years of education, and tear severity are examined and reported as β (95% confidence interval). Standardized deviations at patient and clinic levels describing the variability at the specific level, along with the apparent model performance (R2), are reported. Abbreviations: BMI, body mass index; ASA, American Society of Anesthesiologists.

While an increasing number of years of education was consistently associated with better scores, greater feelings of depression and anxiety, smoking and ASA group III-IV were consistently associated with worse scores (coefficients are shown in Table 2). Tear severity showed a consistent negative association solely for the Constant-Score. Apparent conditional and marginal R^2^ ranged from 0.62 to 0.72 and 0.55 to 0.66, respectively [33].

## Discussion

The ARCR_Pred cohort is the first prospective study following patients after an ARCR in Switzerland in a multicenter setting. The study included 973 patients after screening of 2350 individuals across 19 study centers. Although no major differences in key characteristics between enrolled and non-enrolled patients, the number of patients with massive tears was overrepresented in our study; which may affect the rates of occurrence of post-operative shoulder stiffness (POSS) [34] and re-tear [12]. However, the large proportion of massive tears in our study might be due to the tear severity definition we used from Gerber et al. [23]. Lädermann et al. suggested that tears with retraction should be separately reported, as they more accurately describe tear patterns and predict function [35]. When compared to other similar prospective cohorts initiatives following patients after an ARCR [36], our study showed high follow-up rates reaching 99%, 95%, 89% and 88% at 6 weeks, 6, 12 and 24 months, respectively. When lost to follow-up, patients had slightly lower last recorded outcome values.

Our results showed that functional scores improved over time for Swiss patients undergoing an ARCR. At 6-month, positive change rates ranged between 83% and 92%, increased to 94% to 97% at 12-month follow-up, and remaining high at 24-month (91% to 96%). This is consistent with the results of another recent similar Swiss studies [37, 38]. However, we did not use minimal important changes to interpret our findings. For example, if we considered a change of 4 to 7 points in the OSS being plausible minimal important changes (as recently reported in other studies in the field of ARCR [39] and shoulder arthroplasty [33, 40]), the proportion of minimally improved patients with available follow-up at 24-month would be 92% (N = 782) or 86% (N = 737), respectively. Such threshold values were not yet established for Swiss ARCR patients. There is a need to establish relevant clinimetrics for ARCR patients for interpretation purposes. The subsequent identification of patients not (minimally) benefiting from ARCR procedures in terms of function would then be possible. The development of related clinical prediction models would help in providing patients and surgeons with evidence-based and transparent ARCR success expectations.

Our study found that patient populations differed according to the hospital type, with a higher proportion of highly educated patients in better health status undergoing ARCR in private hospitals. This may be explained by the greater likelihood of these patients holding supplementary hospital insurance, which covers additional services during inpatient stays [17]. The baseline case-mix should be considered for future ARCR outcome comparisons. In our study, we observed inconsistent differences in post-operative functional outcomes, after adjusting for baseline case-mix variables. By focusing on overall function, we provide a different perspective on recent findings supporting that public hospitals have higher retear rates 12 months after ARCR without reporting functional outcomes differences [41]. Moreover, while a recent study found ARCR procedures to be cost-effective in a specialized private orthopedic clinic [37], similar data describing the cost-effectiveness of ARCR are currently lacking at a national level. Potential cost-efficiency differences [42] between hospital type could then be evaluated, particularly given that musculoskeletal disorders are a major driver of healthcare spendings [43], which have nearly doubled in Switzerland over the past 20 years [44].

The results of our study indicate that the number of years of education, anxiety and depression, smoking status, and ASA classification showed consistent associations with modeled outcomes. These variables were already identified as potential prognostic factors for post-operative functional outcomes in our recent prediction model [45] or systematic review works [13]. Finally, while we adjusted for several baseline characteristics, the linear mixed models did not include operation details, surgeon profiles and 6-week post-operative management variables, which is leaving room for potential residual confounding. In case researchers are interested in causal inference, the use of relevant specific methods, such as propensity score matching, would be warranted.

Over the recent years, there has been an increasing interest in defining quality metrics for surgical interventions [46]. Indeed, benchmarking activities could foster a learning collaborative environment [47]. But, to ensure fair comparisons across hospitals, researchers should either focus on a specific subset of patients with similar characteristics or identify a set of case-mix variables that should be accounted for before conducting such analyses. With the present manuscript, we provide Swiss orthopedic surgeons and the literature with important baseline case-mix variables that should be accounted for when performing benchmarking activities.

## Conclusions

The ARCR_Pred study included 973 patients across 19 study centers, with high follow-up rates at 6-, 12- and 24-month. The results of this study appear to be generalizable to the population of patients undergoing ARCR in Switzerland. Functional scores improved over time, but these improvements should be further interpreted in light of minimal important changes, which have not yet been established for Swiss patients. The results of the linear mixed models showed consistent associations between baseline case-mix variables, such as number of years of education, level of depression and anxiety, smoking status or ASA classification and functional outcomes. Private hospitals tended to show better scores at 12-month, but no consistent differences in functional scores at baseline, 6- and 24-month follow-up between private and public hospitals could be identified. The inclusion of (post-)operative variables and surgeon profiles in the models describing functional outcomes is planned for future analyses.

## Supporting information

S1 Table Screened patients key characteristics. (DOCX)

S2 Table Lost-to-follow-up patients key characteristics. (DOCX)

S3 Table Functional outcomes comparison. (DOCX)

S1 File Data sharing statement. (DOCX)
