# Supplementary material for: Baseline characteristics and 2-year functional outcome data of patients undergoing an arthroscopic rotator cuff repair in Switzerland, results of the ARCR_Pred study
Source: PLoS One. 2025 Jan 10;20(1):e0316712. doi: 10.1371/journal.pone.0316712 (PMC11723628; doi:10.1371/journal.pone.0316712)
Supplement: S1 Table — (DOCX) [file pone.0316712.s001.docx]

**Table. Screened patients key characteristics**

| **Characteristic** | **Overall,**  **N = 1,890** | **Not enrolled,**  **N = 917** | **Enrolled,**  **N = 973** | **Difference^1^** |
| --- | --- | --- | --- | --- |
| Age | 58 (10; 22 - 84) | 58 (10; 22 - 84) | 58 (9; 22 - 84) | 0 |
| Male sex | 1,171 (62%) | 560 (61%) | 611 (63%) | 0.04 |
| Tear severity |  |  |  | 0.56 |
| Partial tear | 378 (20%) | 231 (25%) | 147 (15%) |  |
| Single full tear | 514 (27%) | 259 (28%) | 255 (26%) |  |
| Two or three tendons (only one full) | 658 (35%) | 241 (26%) | 143 (15%) |  |
| Massive tear | 340 (18%) | 186 (20%) | 428 (44%) |  |
| Public hospital | 793 (42%) | 350 (38%) | 443 (46%) | 0.15 |
| Mean (SD; min - max); n (%) | | | | |
| 1: Standardized Mean Difference | | | | |
