# Supplementary material for: Baseline characteristics and 2-year functional outcome data of patients undergoing an arthroscopic rotator cuff repair in Switzerland, results of the ARCR_Pred study
Source: PLoS One. 2025 Jan 10;20(1):e0316712. doi: 10.1371/journal.pone.0316712 (PMC11723628; doi:10.1371/journal.pone.0316712)
Supplement: S2 Table — (DOCX) [file pone.0316712.s002.docx]

**Table.** **Lost-to-follow-up patient's key characteristics**

|  | **6-month** | | | | **12-month** | | | **24-month** | | |
| --- | --- | --- | --- | --- | --- | --- | --- | --- | --- | --- |
| **Characteristic** | **Overall,**  **N = 973** | **Not missing,**  **N = 915** | **Missing,**  **N = 58** | **Diff.^1^** | **Not missing,**  **N = 876** | **Missing,**  **N = 97** | **Diff.^1^** | **Not missing,**  **N = 856** | **Missing,**  **N = 117** | **Diff.^1^** |
| **Age** | 57 (9; 21 - 84) | 57 (9; 21 - 84) | 57 (11; 29 - 80) | 0.01 | 57 (9; 21 - 84) | 56 (11; 23 - 80) | 0.09 | 57 (9; 21 - 84) | 56 (10; 25 - 80) | 0.12 |
| **Male sex** | 611 (63%) | 571 (62%) | 40 (69%) | 0.14 | 550 (63%) | 61 (63%) | 0 | 530 (62%) | 81 (69%) | 0.15 |
| **Tear severity** |  |  |  | 0.16 |  |  | 0.26 |  |  | 0.1 |
| *Partial tear* | 147 (15%) | 141 (15%) | 6 (10%) |  | 134 (15%) | 13 (13%) |  | 130 (15%) | 17 (15%) |  |
| *Single full tear* | 255 (26%) | 240 (26%) | 15 (26%) |  | 238 (27%) | 17 (18%) |  | 225 (26%) | 30 (26%) |  |
| *Two or three tendons (only one full)* | 143 (15%) | 134 (15%) | 9 (16%) |  | 125 (14%) | 18 (19%) |  | 122 (14%) | 21 (18%) |  |
| *Massive tear* | 428 (44%) | 400 (44%) | 28 (48%) |  | 379 (43%) | 49 (51%) |  | 379 (44%) | 49 (42%) |  |
| **Public hospital** | 443 (46%) | 404 (44%) | 39 (67%) | 0.48 | 387 (44%) | 56 (58%) | 0.27 | 378 (44%) | 65 (56%) | 0.23 |
| **Oxford Shoulder Score** |  |  |  |  |  |  |  |  |  |  |
| *Baseline* | 27 (9; 0 - 48) | 27 (9; 0 - 48) | 25 (9; 7 - 45) | 0.32 | 27 (9; 0 - 48) | 25 (8; 3 - 43) | 0.26 | 27 (9; 0 - 48) | 27 (8; 3 - 45) | -0.02 |
| *6-month* |  |  |  |  | 40 (7; 6 - 48) | 38 (9; 16 - 48) | 0.25 | 40 (7; 6 - 48) | 37 (8; 8 - 48) | 0.34 |
| *12-month* |  |  |  |  |  |  |  | 43 (7; 8 - 48) | 41 (7; 22 - 48) | 0.36 |
| Mean (SD; min - max); n (%) | | | | | | | | | | |
| 1: Standardized Mean Difference  This table presents the key characteristics of loss-to-follow-up patients and compares them to patients with complete follow-ups. | | | | | | | | | | |
