# Supplementary material for: Baseline characteristics and 2-year functional outcome data of patients undergoing an arthroscopic rotator cuff repair in Switzerland, results of the ARCR_Pred study
Source: PLoS One. 2025 Jan 10;20(1):e0316712. doi: 10.1371/journal.pone.0316712 (PMC11723628; doi:10.1371/journal.pone.0316712)
Supplement: S3 Table — (DOCX) [file pone.0316712.s003.docx]

**Table. Functional outcomes comparison.**

| **Parameter** | **N** | **Overall,**  **N = 973** | **Private,**  **N = 530** | **Public,**  **N = 443** | **Difference** | **95% CI** | |
| --- | --- | --- | --- | --- | --- | --- | --- |
| **Constant-Score (0-100)** |  |  |  |  |  |  | |
| *Baseline* | 970 | 50 (18; 2 - 95) | 51 (18; 2 - 85) | 50 (19; 8 - 95) | 1.4 | -0.93, 3.7 | |
| *6-month* | 891 | 69 (15; 9 - 98) | 72 (14; 9 - 98) | 65 (15; 11 - 96) | 6.7 | 4.7, 8.6 | |
| *Change baseline-6-month* | 888 | 18 (19; -48 - 70) | 20 (19; -47 - 68) | 16 (20; -48 - 70) | 4.8 | 2.2, 7.4 | |
| *12-month* | 849 | 77 (12; 9 - 98) | 80 (10; 23 - 98) | 74 (13; 9 - 98) | 5.7 | 4.1, 7.3 | |
| *Change baseline-12-month* | 847 | 27 (18; -31 - 87) | 29 (17; -16 - 87) | 25 (19; -31 - 76) | 4 | 1.5, 6.5 | |
|  |  |  |  |  |  |  | |
| **Oxford Shoulder Score (0-48)** |  |  |  |  |  |  | |
| *Baseline* | 973 | 27 (9; 0 - 48) | 27 (9; 2 - 48) | 27 (9; 0 - 48) | 0.23 | -0.89, 1.4 | |
| *6-month* | 914 | 40 (8; 6 - 48) | 40 (7; 7 - 48) | 39 (8; 6 - 48) | 0.97 | -0.04, 2.0 | |
| *Change baseline-6-month* | 914 | 12 (9; -25 - 40) | 13 (9; -25 - 40) | 12 (10; -15 - 37) | 0.81 | -0.42, 2.0 | |
| *12-month* | 876 | 43.3 (6.6; 8.0 - 48.0) | 44.1 (5.6; 11.0 - 48.0) | 42.4 (7.5; 8.0 - 48.0) | 1.7 | 0.79, 2.6 | |
| *Change baseline-12-month* | 876 | 16 (9; -14 - 45) | 16 (8; -11 - 45) | 15 (10; -14 - 39) | 1.4 | 0.16, 2.6 | |
| *24-month* | 854 | 44.2 (6.4; 9.0 - 48.0) | 44.8 (5.7; 9.0 - 48.0) | 43.5 (7.2; 12.0 - 48.0) | 1.2 | 0.33, 2.1 | |
| *Change baseline-24-month* | 854 | 17 (9; -16 - 46) | 17 (9; -16 - 46) | 16 (10; -12 - 43) | 1 | -0.29, 2.3 | |
|  |  |  |  |  |  |  |  |
| **Pain (numeric rating scale) (0-10)** |  |  |  |  |  |  |  |
| *Baseline* | 973 | 6 (2; 0 - 10) | 6 (2; 1 - 10) | 6 (2; 0 - 10) | -0.13 | -0.41, 0.14 |  |
| *6-month* | 914 | 3 (2; 0 - 10) | 3 (2; 0 - 10) | 3 (2; 0 - 10) | -0.17 | -0.47, 0.13 |  |
| *Change baseline-6-month* | 914 | -3 (3; -10 - 7) | -3 (3; -10 - 7) | -3 (3; -10 - 6) | 0.01 | -0.34, 0.36 |  |
| *12-month* | 876 | 2 (2; 0 - 10) | 2 (2; 0 - 8) | 2 (2; 0 - 10) | -0.53 | -0.81, -0.25 |  |
| *Change baseline-12-month* | 876 | -4 (2; -10 - 4) | -4 (2; -10 - 4) | -4 (3; -10 - 3) | -0.39 | -0.73, -0.05 |  |
| *24-month* | 856 | 2 (2; 0 - 10) | 2 (2; 0 - 10) | 2 (2; 0 - 9) | -0.11 | -0.40, 0.18 |  |
| *Change baseline-24-month* | 856 | -4 (3; -10 - 5) | -4 (3; -10 - 4) | -4 (3; -10 - 5) | 0.02 | -0.35, 0.38 |  |
|  |  |  |  |  |  |  |  |
| **Subjective Shoulder Value (0-100)** |  |  |  |  |  |  |  |
| *Baseline* | 973 | 47 (19; 0 - 100) | 47 (19; 0 - 100) | 46 (20; 0 - 90) | 1 | -1.4, 3.5 | |
| *6-month* | 913 | 76 (18; 3 - 100) | 78 (17; 3 - 100) | 74 (18; 10 - 100) | 3.7 | 1.4, 6.0 | |
| *Change baseline-6-month* | 913 | 29 (23; -50 - 90) | 30 (23; -50 - 90) | 28 (24; -45 - 90) | 2.7 | -0.37, 5.7 | |
| *12-month* | 875 | 86 (15; 20 - 100) | 88 (13; 20 - 100) | 83 (17; 20 - 100) | 5.1 | 3.1, 7.2 | |
| *Change baseline-12-month* | 875 | 39 (23; -40 - 100) | 41 (22; -40 - 100) | 37 (25; -30 - 95) | 4 | 0.88, 7.1 | |
| *24-month* | 855 | 88 (16; 0 - 100) | 90 (14; 0 - 100) | 86 (17; 1 - 100) | 3.7 | 1.5, 5.9 | |
| *Change baseline-24-month* | 855 | 41 (24; -50 - 100) | 43 (23; -50 - 100) | 40 (24; -40 - 95) | 2.7 | -0.47, 6.0 | |
| Mean (SD; min - max) | | | | | | | |
| This table illustrates the ARCR_Pred patient outcomes stratified by hospital type (private vs. public). It includes measures such as the Constant Score, Subjective Shoulder Value, Oxford Shoulder Score, and Pain Numeric Rating Scale at baseline, 6-month, 12-month, and 24-month follow-ups. Differences between private and public hospitals are presented along with 95% confidence intervals. Abbreviations: CI = Confidence Interval. | | | | | | | |
