# Supplementary material for: Baseline characteristics and 2-year functional outcome data of patients undergoing an arthroscopic rotator cuff repair in Switzerland, results of the ARCR_Pred study
Source: PLoS One. 2025 Jan 10;20(1):e0316712. doi: 10.1371/journal.pone.0316712 (PMC11723628; doi:10.1371/journal.pone.0316712)
Supplement: S1 File — (DOCX) [file pone.0316712.s004.docx]

**S1 File. Data sharing statement**

Due to ethical and legal restrictions imposed by our ARCR_Pred Scientific Board, and to ensure the Principal Investigators have the opportunity to publish key findings, the underlying data set cannot be made publicly available until the end of a period of embargo of two years after the end of the study in September 2024. Following this period, metadata describing the type, size, and content of the dataset will be published along with the study protocol on the open repository Zenodo (<https://zenodo.org/>). The statistical code produced to obtain the results was uploaded to Zenodo (Stojanov, T. (2024). Code for Baseline characteristics and 2-year functional outcome data of patients undergoing an arthroscopic rotator cuff repair in Switzerland, results of the ARCR_Pred study. Zenodo. <https://doi.org/10.5281/zenodo.14234547>) and will be made available upon reasonable request. Researchers wishing to access the code and the full dataset will be able to file a request with the Data Access Committee of the Medical Faculty of the University of Basel (MF-DAC – email: [med-dac@unibas.ch](mailto:med-dac@unibas.ch)). The MF-DAC will act as an independent assessor of the request and grant access to the dataset if all ethical, legal, and scientific conditions are met.
